# ATP-sensitive potassium channels alleviate postoperative pain through JNK-dependent MCP-1 expression in spinal cord

**DOI:** 10.3892/ijmm.2015.2143

**Published:** 2015-03-17

**Authors:** XIANG ZHU, JINQIAN LIU, YONGJING GAO, SU CAO, SHIREN SHEN

**Affiliations:** 1 Department of Anesthesiology, Affiliated Hospital of Nantong University, Nantong, Jiangsu 226001; 2 Department of Anesthesiology, Binzhou Medical University Hospital, Binzhou, Shandong 256603; 3 Institute of Nautical Medicine, Nantong University, Nantong, Jiangsu 226001, P.R. China

**Keywords:** skin/muscle incision and retraction, adenosine triphosphate-sensitive potassium, p-JNK, pinacidil, glibenclamide, astrocytes, chemokines, antinociception

## Abstract

Although adenosine triphosphate-sensitive potassium (KATP) channels have been proven to be involved in regulating postoperative pain, the underlying mechanism remains to be investigated. In this study, we aimed to determine the role of spinal KATP channels in the control of mechanical hypersensitivity in a rat pain model, in which rats were subjected to skin/muscle incision and retraction (SMIR) surgery, as well as in LPS-stimulated astrocytes. The results showed that KATP channel subunits Kir6.1, SUR1 and SUR2 were normally expressed in the spinal cord and significantly downregulated after SMIR. SMIR caused a marked increase in monocyte chemoattractant protein-1 (MCP-1) mRNA expression and in the protein level of p-JNK in the spinal cord. Intrathecal administration of a KATP channel opener pinacidil (Pina) suppressed mechanical allodynia after SMIR and significantly downregulated the MCP-1 mRNA expression and the protein level of p-JNK induced by SMIR. Inverted fluorescence microscopy showed that Kir6.1 was co-localized with astrocytes only and SUR2 was co-localized primarily with neurons, in a small amount with astrocytes. Furthermore, *in vitro* studies showed that following incubation with LPS, the astrocytic MCP-1 mRNA expression and p-JNK content were markedly increased, whereas the mRNA levels of Kir6.1 and SUR2 were significantly downregulated in astrocytes. KATP channel opener pinacidil inhibited the LPS-triggered MCP-1 and p-JNK elevation in rat primary astrocytes. The results suggested that KATP channel opener treatment is an effective therapy for postoperative pain in animals, through the activation of the JNK/MCP-1 pathway in astrocytes.

## Introduction

Postoperative pain is a common clinical problem encountered in patients undergoing thoracotomy, inguinal hernia repair, living donor nephrectomy, amputation, gynecological surgery, and gastro-intestinal surgery (1). Postoperative pain can be resolved after an initial phase or transit into chronic postoperative pain that seriously affects quality of life and productivity of patients, including a number of negative effects on mood, daily activities, sleep, cognitive function, and social life (2,3). Thus, there is a need for therapeutic approaches to prevent chronic postoperative pain.

Mounting evidence has demonstrated that adenosine triphosphate-sensitive potassium (KATP) channels regulate nociception. For example, Zoga *et al* (4) reported that KATP channel subunits SUR1, SUR2 and Kir6.2, but not Kir6.1, were expressed in rat dorsal root ganglion (DRG) neurons, peripheral nerve fibers, glial satellite and Schwann cells. KATP channels were downregulated in DRG neurons and Schwann cells following painful axotomy, suggesting that loss of KATP currents in the DRG neurons may contribute to neuropathic pain (4). Wu *et al* (5) found that the KATP channel subunits SUR1, SUR2 and Kir6.1 but not Kir6.2 were normally expressed in the spinal cord and significantly downregulated after nerve injury. Furthermore, nerve injury-induced downregulation of the KATP channels in the spinal cord may interrupt the astroglial gap junctional function and contribute to neuropathic pain. The KATP channels opener cromakalim may reduce neuropathic pain, probably partly by regulating the astroglial gap junctions (5). Xia *et al* demonstrated that the expression level of KATP channel subunit Kir6.2 in the spinal cord was reduced in bone cancer pain. Activation of KATP channels by the KATP channels opener pinacidil (Pina) at the spinal level reduced pain hypersensitivity associated with bone cancer pain (6). The abovementioned studies suggested that the expression pattern of KATP channel subunits in the spinal cord remains controversial and the role of KATP channels in regulating spinal nociceptive transmission remains to be elucidated.

In this study, we aimed to investigate alterations of the protein expression for KATP channel subunits in the spinal cord after skin/muscle incision and retraction (SMIR), a new model that accurately reflects the clinical scenario of postoperative pain (7). In addition, we assessed the association between KATP channels and the chemokine monocyte chemoattractant protein-1 (MCP-1) as recent findings showed that MCP-1 is also activated in the spinal cord and contributes to the development of inflammatory and neuropathic pain hypersensitivity (8,9).

## Materials and methods

### Animals and grouping

Adult male Sprague-Dawley rats (200–250 g) were purchased from the Experimental Animal Center of Nantong University and kept in the animal housing facility with controlled room temperature (23±1°C) and unlimited access to food and water. The rats were allowed to habituate to the housing facility for 3 days before the experiments were initiated. Surgical and experimental procedures were approved by the Animal Use and Care Committee for Research and Education of Nantong University. Animal treatments were performed according to the Guidelines of the International Association for the Study of Pain (10).

Rats were randomly and evenly divided into 6 groups (n=5): i) normal group, ii) sham-operated group, iii) SMIR model group, iv) SMIR + PBS group, v) SMIR + KATP channels opener Pina group and vi) SMIR + Pina + KATP channel blocker glibenclamide (Gli) group.

SMIR surgery was performed on rats as previously described (7). Briefly, the animals were anesthetized with intraperitoneal injection of pentobarbital sodium (50 mg/kg) and placed in the supine position. After the medial thigh on the right leg was shaved and sterilized, a 1.5–2 cm skin incision, ~4 mm medial to the saphenous vein, was made to expose the muscle of the thigh. A 7–10 mm incision, ~4 mm medial to the saphenous nerve, was made in the superficial (gracilis) muscle layer of the thigh. The superficial muscle was further isolated by spreading blunt scissors within the muscle incision site to allow the insertion of a micro-dissecting retractor. The retractor was inserted into the incision site, and the superficial muscle of the thigh was retracted by 2 cm. In the period of retraction, the saphenous nerve was displaced and potentially stretched around the retractor, but not compressed against a hard surface such as bone. The animals were covered with a heavy absorbent bench underpad to prevent surgical site dehydration. After 1 h, the muscle and skin of the surgical site was closed with 4.0 Vicryl^®^ sutures. Sham-operated rats underwent the same procedure with the exception of the skin/muscle retraction.

Phosphate-buffered saline (PBS), Pina (4, 20 or 40 *μ*g; Research Biochemicals International, Natick, MA, USA), or Pina (20 *μ*g) + Gli (20 or 50 *μ*g; Sigma, St. Louis, MO, USA) were intrathecally injected at 7 days after SMIR surgery. For intrathecal injection, the animals were anesthetized with isoflurane. The spinal cord puncture was made with a 30-gauge needle between the L4 and L5 level to deliver the reagents (40 *μ*l) to the cerebral spinal fluid. Immediately after the needle entry into the subarachnoid space, a brisk tail flick was observed (11).

### Cell culture and treatment

Primary astrocytes were prepared from cerebral cortexes of neonatal rats (postnatal day 1, P1). The cerebral hemispheres were isolated and transferred to ice-cold Hank’s buffer (Invitrogen, Carlsbad, CA, USA), and the meninges were carefully removed. Tissues were then minced into 1-mm sections, triturated, filtered through a 100-*μ*m nylon screen, and collected by centrifugation at 3,000 × g for 5 min. The cell pellets were re-suspended in a medium containing 10% fetal bovine serum in low-glucose DMEM. After filtration through a 10-*μ*m screen, the cells were plated in 6-well plates at a density of 2.5×10^5^ cells/cm^2^ and cultured for 10–12 days. The medium was replaced twice a week. When the cells grew to 95% confluence (10–12 days), 15 mM dibutyryl cyclic adenosine monophosphate (cAMP) (Sigma-Aldrich) was added to induce differentiation. The cells were used 3 days later.

When the cells were ready, they were randomly divided into four groups: i) normal control; ii) LPS treatment group: the astrocytes were stimulated with LPS (1 *μ*g/ml) for 3 h in a 37°C incubator; iii) Pina group: the astrocytes were pre-incubated with Pina (200 mM) for 30 min; iv) Pina + LPS group: the astrocytes were pre-incubated with Pina (200 mM) for 30 min, followed by incubation with LPS for 3 h; v) Pina + Gli + LPS group: the cells were pre-incubated with Pina (200 mM) + Gli (50 or 500 mM) for 30 min and subsequently exposed to LPS for 3 h; vi) Gli group: the astrocytes were pre-incubated with Gli (200 mM) for 30 min. In addition, to examine whether the expression of p-JNK was dependent on exposure time, the cells were treated with 1 *μ*g/ml LPS for 15, 30 and 60 min, respectively, following treatment with d-cAMP for 72 h. After the above treatments, the cells were collected for RT-PCR and western blotting.

### Measurement of mechanical allodynia

Mechanical allodynia was assessed using Up-Down paradigm (12) with von Frey filaments (IITC Life Science Inc., Victory Blvd, Woodland Hills, CA, USA) ranging from 1.4 to 26 g. Animals were placed on an elevated wire mesh floor and confined underneath individual overturned Perspex boxes (26×20×14 cm). Tests were conducted in the morning between 8:30 and 11:30 a.m. A series of von Frey hair stimuli was delivered in an ascending order of forces to the mid-plantar area of the hindpaw encircled by tori/footpads. The responses were recorded in grams of paw withdrawal averaged over 3–5 applications referred to as mechanical withdrawal threshold (MWT). Behavioral tests were performed prior to, and 1, 3, 5, 7, 10, 21, 28 and 32 days following SMIR surgery.

### Reverse-transcriptase polymerase chain reaction (RT-PCR)

At 3 and 7 days after SMIR surgery, the animals were terminally anesthetized with pentobarbital (40 mg/kg, intraperitoneal) and transcardially perfused with PBS at room temperature. The L3-L5 spinal cord segments from each rat were dissected. Total RNA was extracted from the spinal cord or cultured astrocytes using TRIzol reagent (Invitrogen, Carlsbad, CA, USA). Total RNA (1 *μ*g) was reverse-transcribed to cDNAs using PrimeScript reverse transcriptase (Takara, Kyoto, Japan). The *MCP-1*, *Kir6.1*, *Kir6.2*, *SUR1*, and *SUR2* genes were amplified from the cDNA using the Rotor-Gene 3000 real-time DNA analysis system (Corbett Research, Sydney, Australia). The detailed primer sequences for each gene are provided in Table I. Amplification conditions and incubation conditions were as follows: 30 sec at 50°C, then thermo-cycling for 40 cycles of 5 sec at 95°C, 45 sec at 60°C, then 20 sec at 72°C. The threshold cycle number and reaction efficiencies of each condition were identified with Rotor-Gene Analysis Software 6.1 (Corbett Research). The mean relative mRNA levels were determined with at least three separate reactions per condition. Melt curves were performed on completion of the cycles to ensure that non-specific products were absent. Quantification was performed by normalizing cycle threshold (Ct) values with glyceraldehyde-3-phosphate dehydrogenase (*GAPDH*) Ct and analyzed with the 2^−ΔΔCt^ method.

### Western blotting

Tissue samples of the spinal cord at each time point after SMIR were prepared in the same manner as for PCR. Spinal cord tissues or astrocytes were homogenized in a lysis buffer containing protease and phosphatase inhibitors (Sigma). Protein concentrations were determined by the BCA Protein Assay (Pierce, Rockford, IL, USA). Protein (30 *μ*g) was loaded for each lane, separated by SDS-polyacrylamide gel electrophoresis (SDS-PAGE-10%), and transferred to polyvinylidene difluoride membranes. After the transfer, the membranes were blocked with 5% milk in PBS with 0.1% Tween-20 for 2 h at room temperature, and incubated overnight at 4°C with polyclonal antibody against Kir6.1 (rabbit, 1:200, Sigma), Kir6.2 (goat, 1:200, Santa Cruz Biotechnology Inc., Santa Cruz, CA, USA), SUR1 (rabbit, 1:200, Santa Cruz Biotechnology Inc.), SUR2 (goat, 1:200, Santa Cruz Biotechnology Inc.), and p-JNK (rabbit, 1:2,000, Santa Cruz Biotechnology Inc.). For the loading control, the blots were also probed with GAPDH antibody (mouse, 1:20,000, Millipore, Billerica, MA, USA). The membranes were washed three times with TBST buffer and incubated with the secondary antibody (1:2,000) for 2 h followed by three washings. Blots were visualized in ECL solution and exposed on hyperfilms (Bio-Rad, Hercules, CA, USA) for 2–5 min. Specific bands were then evaluated by apparent molecular size. The intensity of the selected bands was analyzed using Image J software (NIH, Bethesda, MD, USA).

### Immunohistochemistry

On day 0 and day 7 after SMIR, the rats were anesthetized with pentobarbital and perfused transcardially with PBS followed by 4% paraformaldehyde in PBS (250 ml; pH 7.0). After the perfusion, the L3-L5 spinal cord from each rat was extracted and post-fixed in the same fixative at 4°C overnight and then placed in 20% and subsequently in 30% sucrose solution at 4°C overnight, respectively. After cryoprotection with 30% sucrose, the spinal cord was cut at 30-*μ*m on a freezing microtome. The sections were first blocked with 5% goat serum for 2 h at room temperature and then incubated with the following primary antibodies: Kir6.1 (rabbit, 1:80, Sigma), Kir6.2 (goat, 1:80, Santa Cruz Biotechnology Inc.), SUR1 (rabbit, 1:50, Santa Cruz Biotechnology Inc.), SUR2 (goat, 1:100, Santa Cruz Biotechnology Inc.), glial fibrillary acidic protein (GFAP) antibody (mouse, 1:5,000, Millipore), OX-42 antibody (mouse, 1:5,000, Serotec, Kidlington, Oxford, UK), and NeuN antibody (mouse, 1:5,000, Millipore) at 4°C overnight. The sections were then incubated for 2 h at room temperature with Cy3- or FITC-conjugated secondary antibodies (1:400, Jackson ImmunoResearch, West Grove, PA, USA). For double immunofluorescence, the sections were incubated with a mixture of mouse and rabbit (or goat) primary antibodies followed by a mixture of FITC- and CY3-conjugated secondary antibodies. The stained sections were examined with a Leica fluorescence microscope, and images were captured with a CCD Spot camera. The sections with double staining were imaged with an FV10i confocal microscope (Olympus, Tokyo, Japan).

### Statistical analysis

Data were presented as mean ± SEM and analyzed by the SPSS 16.0 (SPSS Inc., Chicago, IL, USA). For behavioral studies, the data were analyzed with two-way analysis of variance followed by a Bonferroni’s test for post-hoc analysis. Differences in gene and protein expression between groups were compared using one-way ANOVA followed by Newman-Keuls post-hoc test or using the Student’s t-test if only two groups were applied. P<0.05 was considered statistically significant.

## Results

### SMIR surgery induces persistent significant mechanical hypersensitivity

**Figure 1:**
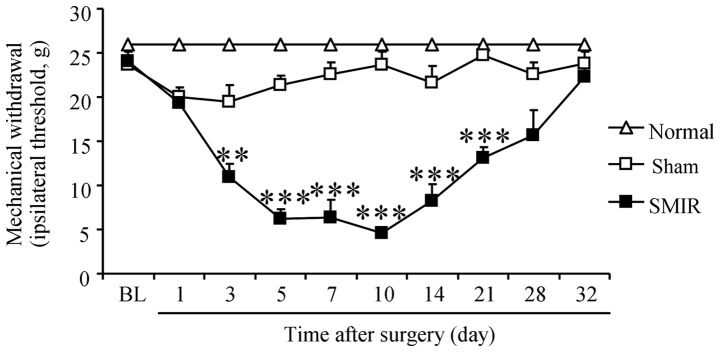
SMIR surgery induces persistent significant mechanical hypersensitivity. ^**^P≤0.01; ^***^P≤0.001 vs. BL. SMIR, skin/muscle incision and retraction BL, baseline.

In this study, we established the postoperative pain model by using a retractor to properly open the skin and superficial muscle for 1 h. As SMIR did not induce significant heat hyperalgesia or cold allodynia (13), mechanical hypersensitivity became the focus. No significant differences in basal MWT between the normal, SMIR and sham-operated groups were identified. However, the MWT of SMIR-operated rats was decreased at postoperative day 1 and this decrease was maintained for >21 days. During these periods, SMIR-induced mechanical hypersensitivity in the ipsilateral paw was significant at postoperative day 3 (P<0.01) and most prominent at postoperative day 10 compared with the baseline (Fig. 1). However, the sham-operated and normal groups did not show any marked changes at any of the time points, and no significant difference was observed between the sham and normal groups (P>0.05). These results indicated that SMIR induced persistent significant mechanical hypersensitivity.

### Decrease in KATP channel subunits and increase in MCP-1 expression after SMIR in the spinal cord

**Figure 2:**
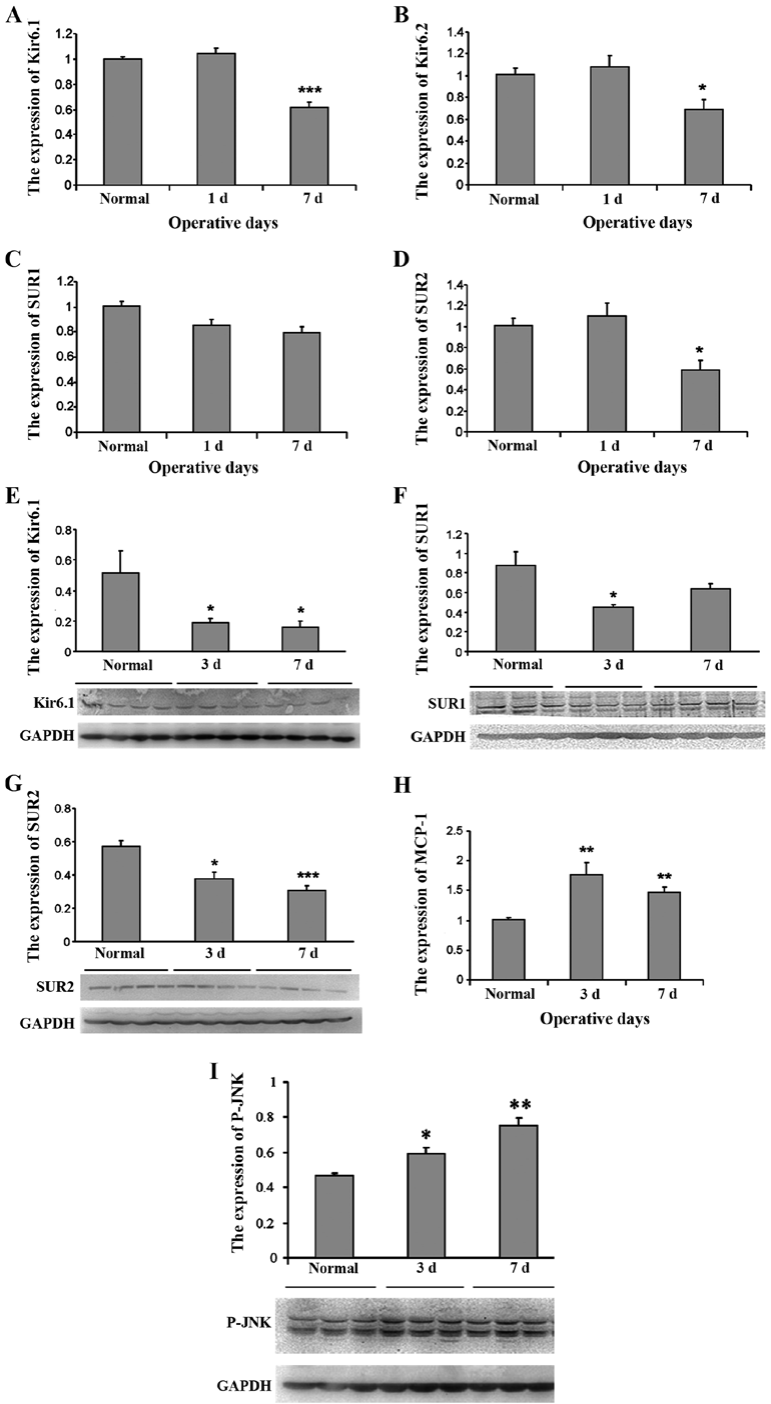
Decrease of KATP channel subunits and increase of MCP-1 and JNK expression after SMIR in the spinal cord KATP channel subunits. (A–D) mRNA expression of KATP channel subunits. (E–G) Protein expression of KATP channel subunits. (H) mRNA expression of MCP-1. (I) Protein expression of JNK. *P≤0.05, ^**^P≤0.01, ^***^P≤0.001 vs. normal. KATP, adenosine triphosphate-sensitive potassium; MCP-1, monocyte chemoattractant protein-1; SMIR, skin/muscle incision and retraction.

To determine whether KATP plays a role during the development of postoperative pain, we first performed a time-course analysis of the subunits of KATP expression in the spinal cord after the SMIR operations. The results showed that compared with normal rats, SMIR caused a significant decrease in the expression of the KATP channel subunits Kir6.1 (Fig. 2A), Kir6.2 (Fig. 2B) and SUR2 (Fig. 2D) on postoperative day 7 (P<0.05, Fig. 2). However, from days 3 to 7 after SMIR, no significant variation of SUR1 mRNA expression was observed compared with the normal rats (P>0.05, Fig. 2C). The ressults from western blot analysis revealed that the expression of the KATP channel subunits Kir6.1 (Fig. 2E) and SUR2 (Fig. 2E) in the spinal cord was significantly decreased at 3 and 7 days after SMIR (P<0.05). SUR1 expression was reduced at post-operative day 3, but returned to the level of the control at post-operative day 7 (Fig. 2F), indicating it may not be important for pain hypersensitivity. The protein expression for Kir6.2 subunit was extremely low and not detectable in the spinal cord. By contrast, the PCR results showed that MCP-1 expression in the spinal cord was significantly increased at 3 and 7 days (P<0.01) after SMIR (Fig. 2H).

### Intrathecal administration of Pina inhibits mechanical allodynia after SMIR and downregulates MCP-1 expression

**Figure 3:**
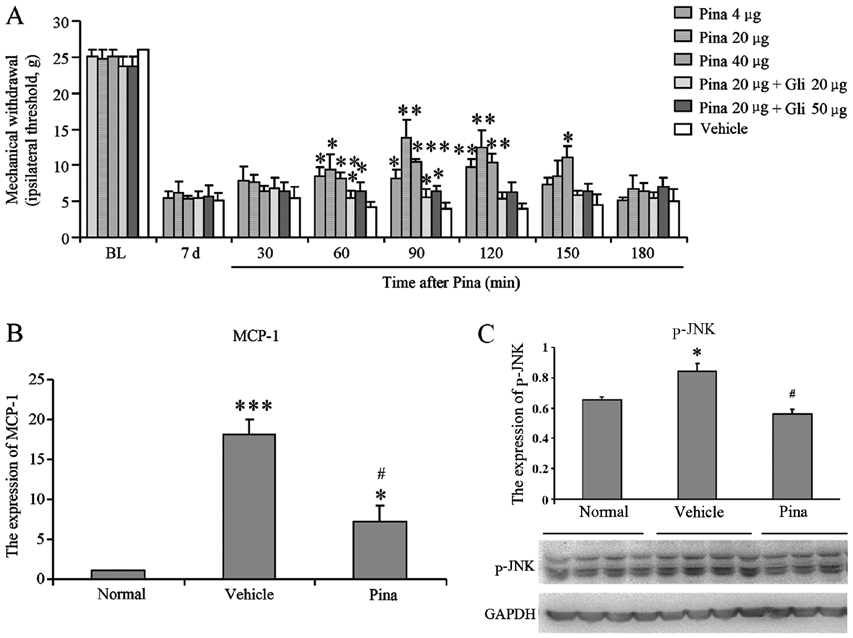
Intrathecal administration of KATP channel opener Pina inhibits mechanical allodynia after SMIR, downregulates MCP-1 expression and JNK activation. (A) Mechanical allodynia. (B) mRNA expression of MCP-1. (C) Protein expression of JNK. ^*^P≤0.05, ^**^P≤0.01, ^***^P≤0.001, vs. normal, ^#^P≤0.05 vs. vehicle. KATP, adenosine triphosphate-sensitive potassium; SMIR, kin/muscle incision and retraction; MCP-1, monocyte chemoattractant protein-1.

SMIR produced long-lasting mechanical allodynia associated with downregulation of the KATP channel subunits SUR2 and Kir6.1. We determined whether a KATP channel opener was capable of reducing the allodynia. Therefore, we examined the analgesic effect of KATP channel opener Pina by intracisternal injection at 7 days after SMIR. As shown in Fig. 3A, intracisternal injection of Pina markedly increased MWT initiation within 1 h, peaked at 2 h, with the pain returning within 3 h. In addition, the anti-nociceptive effect of 20 *μ*g Pina was the optimum. Therefore, we utilized the 20 *μ*g Pina to determine whether the anti-nociceptive effects of the KATP activators could be relieved by the KATP blocker Gli (20 or 50 *μ*g). As expected, injection of Gli together with the Pina reversed the anti-mechanical nociceptive effects of Pina after 1, 1.5 and 2 h (Fig. 3A).

To determine the possible role of MCP-1 in the KATP opener-induced inhibition of neuropathic pain, we then tested the MCP-1 expression after Pina (20 *μ*g) treatment at 7 days after SMIR. The results indicated Pina significantly reduced SMIR-induced MCP-1 mRNA in the spinal cord (Fig. 3B). These results suggested that opening the KATP channel downregulated MCP-1 expression to alleviate postoperative pain.

### KATP channel opener Pina prevents SMIR-induced JNK activation in spinal cord

Previously it was shown that c-jun-N-terminal kinase (JNK) is an important intracellular kinase and plays a crucial role in the pathogenesis of chronic pain by upregulating MCP-1 expression in spinal cord astrocytes (14). To investigate whether the effect of Pina on SMIR-induced MCP-1 expression was mediated through the JNK pathway, we first examined the activation of JNK by western blotting with phospho-specific antibody at 3 and 7 days after SMIR. The result showed that SMIR induced a persistent p-JNK increase in the spinal cord peaking at day 7 (Fig. 2I). However, intrathecal administration of the KATP channel opener Pina blocked the SMIR-induced upregulation of p-JNK protein, reaching the level of the normal group (Fig. 3C). These results suggested that the JNK signaling pathway was involved in KATP channel in postoperative pain transmission.

### Cellular co-localization of Kir6.1 and SUR2 in the spinal cord

**Figure 4:**
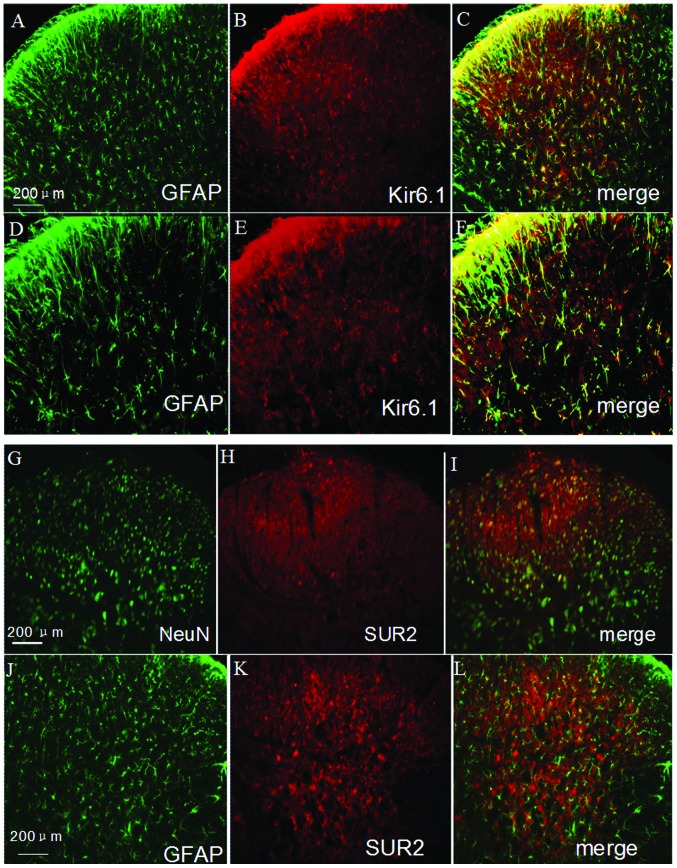
Cellular co-localization of Kir6.1 and SUR2 in spinal cord. Cellular co-localization of (A–F) Kir6.1 and (G-L) SUR2.

Expression of Kir6.2 was not detected from the spinal cord and SMIR did not cause any change in SUR1 expression at postoperative days 1 and 7. Thus, Kir6.1 and SUR2 are involved in post-operative pain. To define the cell distribution of Kir6.1 and SUR2, we performed double staining of Kir6.1 and SUR2 with different cell markers. As shown in Fig. 4, Kir6.1 was primarily co-localized with the astrocytic marker GFAP, but not with neuronal marker NeuN or microglial marker Iba-1, suggesting Kir6.1 is primarily induced in astrocytes in the spinal cord. SUR2 was co-localized with NeuN, while occasionally it was identified in a small amount with GFAP.

### KATP channel opener inhibits LPS-triggered MCP-1 elevation in rat primary astrocytes

**Figure 5:**
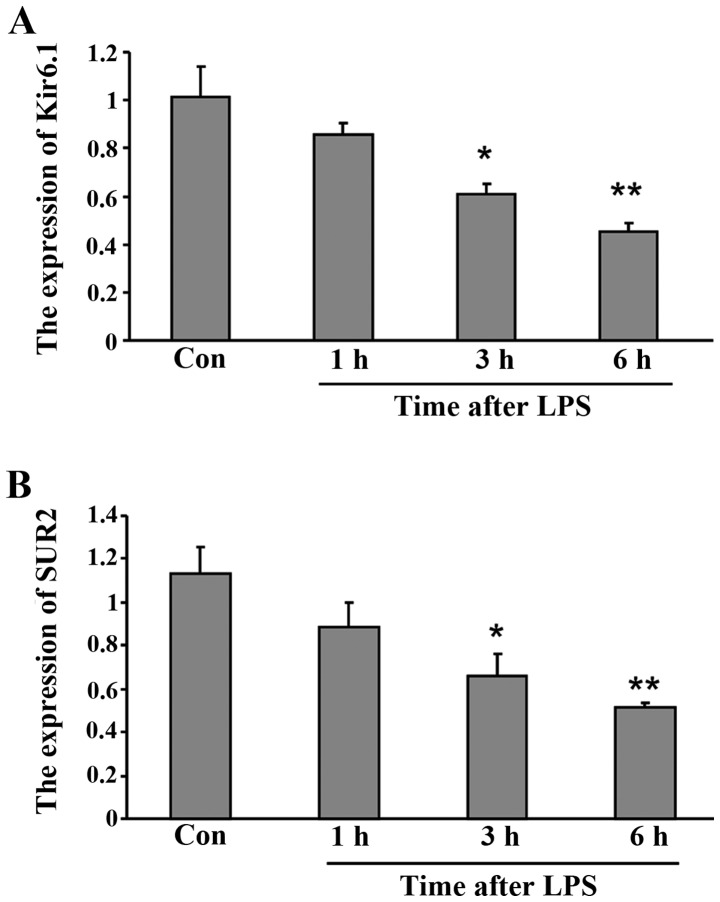
KATP subunit expression after LPS incubation in astrocytes. ^*^P≤0.05, ^**^P≤0.01 vs. con. KATP, adenosine triphosphate-sensitive potassium.

As Kir6.1, SUR2 and MCP-1 are expressed in astrocytes, we determined the interaction of KATP and MCP-1 *in vitro*, and prepared primary astrocytes from cerebral cortexes of neonatal rats. Since astrocytes were known to be activated by inflammatory mediators, we simulated astrocyte activation *in vitro* with LPS, a critical trigger, for inflammatory responses (15). As shown in Fig. 5C, after incubation with LPS (1 *μ*g/ml) for 3 h, astrocytic MCP-1 expression was markedly increased (P<0.01) compared with the control group. By contrast, Kir6.1 and SUR2 expression was significantly decreased when astrocytes were exposed to LPS for 3 and 6 h, respectively (Fig. 5A and 5B).

**Figure 6:**
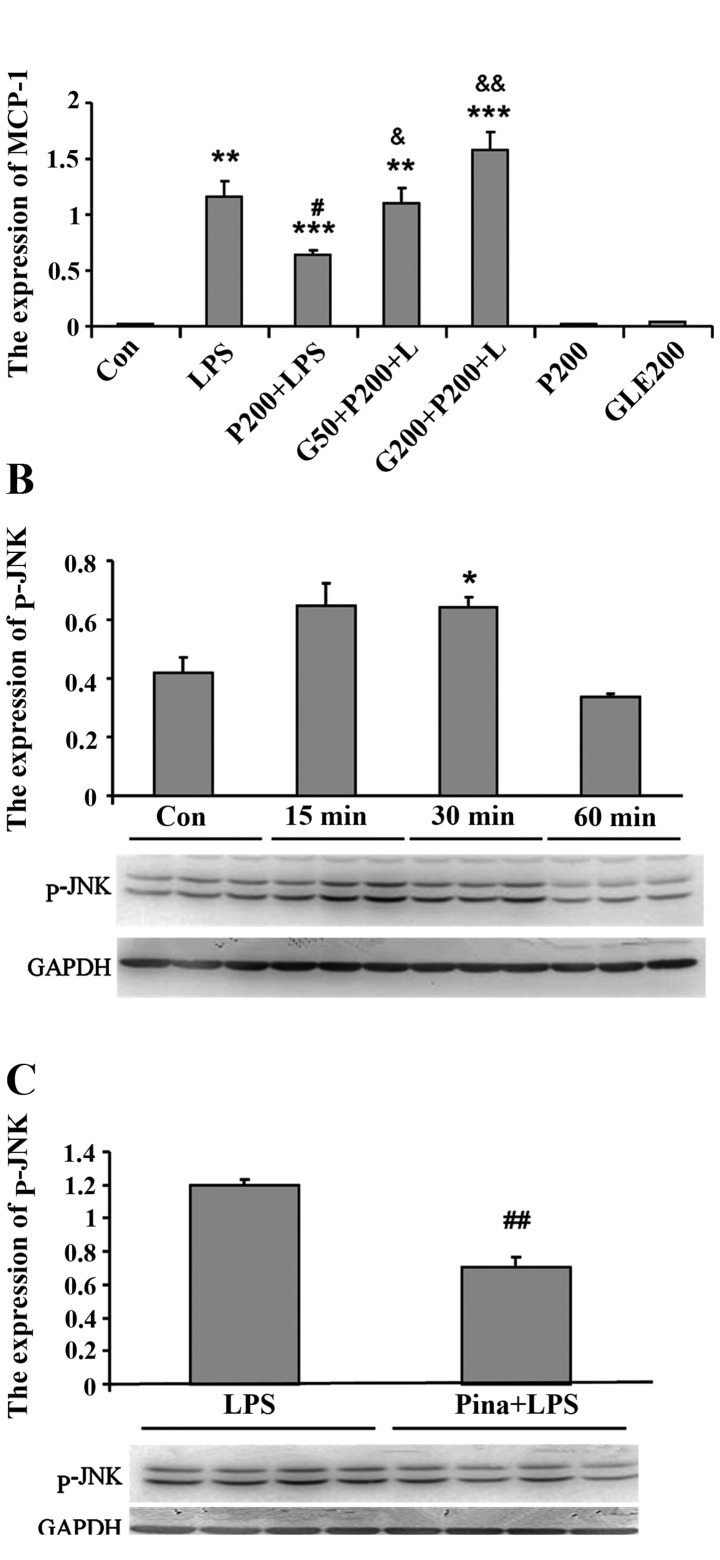
KATP agonist reduces LPS-induced MCP-1 and p-JNK expression in astrocytes. (A) MCP-1 expression. (B and C) p-JNK expression. ^*^P≤0.05, ^**^P≤0.01, ^***^P≤0.001 vs. con; ^#^P≤0.05, ^##^P≤0.01 vs. LPS; ^&^P≤0.05, ^&&^P≤0.01 vs. P200 + LPS. KATP, adenosine triphosphate-sensitive potassium; MCP-1, monocytes chemoattractant protein-1.

To examine the involvement of KATP in MCP-1 expression, we pre-incubated astrocytes with Pina or Pina + Gli for 30 min followed by incubation with LPS for 3 h. As shown in Fig. 5C, Pina significantly alleviated MCP-1 upregulation in LPS co-treated astrocytes (P<0.01) and Gli significantly reversed the inhibition of Pina to MCP-1 expression (P<0.01). Pina or Glib alone had no effect on MCP-1 expression (Fig. 6A). These findings demonstrated that the protective role of Pina in astrocytes was actually due to the opening of the KATP channel.

### KATP agonist reduces the LPS-induced protein level of p-JNK in primary cultured astrocytes

Mounting evidence suggests that JNK is important in mediating the effects of LPS on MCP-1 production (16). Thus, we investigated whether the KATP channel opener affected LPS-induced JNK phosphorylation in astrocytes. After LPS exposure, there was a rapid activation (phosphorylation) of JNK. The p-JNK induction was initiated at 15 min, reached a peak at 30 min, and decreased at 60 min, indicating that the JNK signaling pathway was activated in response to the LPS treatment in astrocytes (Fig. 6B). We detected whether the KATP channel opener regulated LPS-induced JNK phosphorylation at the 30-min time point. Pretreatment of cells with Pina (200 M) decreased LPS-induced increase in phospho-JNK >40 and >50% (Fig. 6C).

## Discussion

Ion channels play a vital role in pain signal initiation and conduction (5). The transient receptor potential channel family and voltage gated sodium channels are among the most intensively studied ion channels in pain signaling (17,18). Increasing attention has been paid to the role of potassium (K^+^) channels in pain (19,20). K^+^ channels play an essential role in setting the resting membrane potential and in controlling the excitability of neurons. Thus, K^+^ channels are potentially attractive peripheral targets for the treatment of pain. One K^+^ channel that is known to regulate excitability in a variety of central and peripheral neurons is the M channel. Another family of K^+^ channels indicated in pain responses is the ATP-sensitive potassium channel (KATP) family (21). The KATP channels play critical roles in regulating membrane excitability and neurotransmitter release, and providing neuroprotection (22). The structure of the KATP channel was initially determined in pancreatic b-cells to be an octameric complex of two types of subunit: pore-forming Kir6.2 and the sulfonylurea receptor SUR1, which belong to the ATP-binding cassette superfamily (23). Additional Kir and SUR subunits were subsequently identified to form complexes with distinct pharmacological properties: the cardiac and skeletal muscle type composed of Kir6.2 and SUR2A, an isoform of SUR1 (24), and the vascular smooth muscle type composed of Kir6.1 and SUR2B, a splice variant of SUR2A. Previous findings have shown that the KATP channel subunits SUR1, SUR2, and Kir6.2, but not Kir6.1, are expressed in rat DRG neurons and in the satellite glial cells. After painful axotomy, KATP channels were downregulated in DRG neurons and Schwann cells (4), and loss of the KATP current may contribute to neuropathic pain through increased membrane excitability and amplified neurotransmitter release (25). Nevertheless in the spinal cord, the protein expression of Kir6.2 was extremely low and not detectable, which is different from that in DRG. RT-PCR results revealed mRNA encoding all KATP channel subunits. However, immunostaining or western blotting did not detect any Kir6.2 at the protein level. This may be attributed to the fact that immunohistochemistry and western blotting were not sufficiently sensitive to detect Kir6.2 protein in the spinal cord, or that Kir6.2 mRNA is not translated into protein in these cells (26). However, the same antibodies did not fail to detect Kir6.2 in DRG, indicating competence of our antibodies, and, therefore the lack of translation of Kir6.1 mRNA to protein in the spinal cord. This finding suggested SUR1 or SUR2 subunits always co-assemble with Kir6.1 or Kir6.2 subunits into functional KATP channel (27). Additionally, the different expression of KATP channels in DRG and the spinal cord may suggest different roles of these channels in DRG and the spinal cord. However, this needs to be further investigated. Our findings from these experiments emphasize the presence of KATP channels in the spinal cord are of the Kir6.1/SUR1 and Kir6.1/SUR2 subtype complex. Spinal administration of a KATP channel opener Pina can prevent or reduce hyperalgesia and allodynia, but did not affect the mechanical sensitivity in vehicle animals. The KATP blocker Gli co-applied with Pina can reverse the effect of analgesic action by Pina, suggesting the Kir6.1/SUR1 and Kir6.1/SUR2 subtype in the spinal cord may be involved in the postoperative pain mechanism and act as an early sensor of stress conditions. On the other hand, the KATP blocker Gli did not affect any of the nocifensive behavior in the absence of KATP activators. This result suggests that KATP does not necessarily play a role in the nociception induced by these stimuli, merely that the existence of these channels provides a means of reducing hyperexcitability when KATP activity is enhanced (28).

Our results show that Kir6.1 was co-localized with astrocytes only and SUR1 and SUR2 were co-localized primarily with neurons, in a small amount with astrocyte. It is now widely recognized that activation of spinal glial cells, including microglia and astrocytes, are involved in central sensitization and mechanical hypersensitivity in acute and persistent pain states (29–31). Astrocytes are recognized as important contributors in pathological pain creation and maintenance (32,33). In normal conditions, astrocytes are relatively resting or quiescent. However, after injury or under disease conditions, they can be converted to reactive states and participate in the pathogenesis of neurological disorders (34,35). Astrocytes are also capable of monitoring changes in synaptic activity and of spatially integrating this information for signaling microvascular units, in particular via dynamic Ca^2+^ signaling between astrocytes and neurons or the endothelium via gap junctions and purinergic transmission. Notably, astrocytes make very close contacts with synapses and astrocyte reaction is more persistent following nerve injury, arthritis, and tumor growth than microglial reaction, exhibiting a better correlation with chronic pain behaviors (32). A close contact with neurons and synapses makes it possible for astrocytes to support and nourish neurons, and regulate the external chemical environment of neurons during synaptic transmission. Accumulating evidence indicates that activated astrocytes can release gliotransmitters such as ATP, glutamate, growth factors, pro-inflammatory cytokines, and chemokines in the spinal cord to enhance and prolong persistent pain states (8,9). MCP-1, also known as monocyte chemotactic protein-1 (MCP-1), is a small 14-kDa protein that signals through the G-protein-coupled receptor CCR2. MCP-1, similar to other chemokines, was initially identified as an immunomodulatory factor that regulates activation and migration of peripheral immune cells. It has been shown to have a neuromodulatory role in spinal nociceptive processing. Spinal nerve ligation induced persistent neuropathic pain and MCP-1 upregulation in the spinal cord (14). MCP-1 contributes to the maintenance of mechanical hypersensitivity after plantar incision and establishes a role for neural glial signaling in postoperative pain (36). Consistent with the abovementioned studies, our results show that the expression of mRNA for MCP-1 levels was significantly increased at 3 and 7 days compared with the normal group in rat after SMIR.

KATP channels and chemokine MCP-1 are expressed in astrocytes and altered in the spinal cord by SMIR, and both of them contribute to postoperative pain. We examined whether there was a connection between them. Intrathecal administration of a KATP channel opener Pina was applied and the results showed that Pina significantly downregulated the expression of MCP-1 mRNA. In order to further verify these results, we prepared primary astrocytes from cerebral cortexes of neonatal rats. We detected MCP-1 production in astrocyte lysates and MCP-1 release in the culture medium. In the non-stimulated conditions, astrocytes expressed low levels of MCP-1. Exposure to LPS-induced rapid and time-dependent increased MCP-1 expression, whereas the mRNA level of Kir6.1 and SUR2 were significantly downregulated in astrocytes. The KATP channel opener inhibited LPS-triggered MCP-1 elevation in rat primary astrocytes. These results show KATP is involved in MCP-1 release in the astrocytes *in vitro* and *in vivo*. However, how KATP regulates the MCP remains to be determined.

Previous findings suggest that activation of the JNK/MCP-1 pathway in astrocytes is important in promoting neuropathic pain (14). Pre-incubation with Gli can prevent the activation of SAPK/JNK (37). Moreover, diazoxide suppressed rotenone-induced mitochondrial membrane potential loss and p38/c-Jun N-terminal kinase activation in microglia, which may, in turn, regulate the production of pro-inflammatory factors (38). Those findings demonstrate a possible link between the KATP channel and the downstream mechanisms of MCP-1 expression in the spinal cord through p-JNK-dependent manner. As expected, we proved that the KATP channel opener inhibited the protein level of p-JNK elevation in rat primary astrocytes, as well as in spinal cord induced by SMIR.

In conclusion, this study has demonstrated that KATP channel opener treatment is an effective approach to relieving postoperative pain. This effect may be mediated by the activation of the JNK/MCP-1 pathway in astrocytes in the spinal cord. As KATP channels may have diverse functional roles in astrocytes and neurons in the spinal cord, further studies are required to explore the potential of KATP channels as targets of therapy against postoperative pain and neurodegeneration.

## Figures and Tables

**Table I:** The detailed primer sequences for the six genes.

Gene	Primer sequences
*GAPDH*	F: 5′-TCC TAC CCC CAA TGT ATC CG-3′
	R: 5′-CCT TTA GTG GGC CCT CGG-3′
*MCP-1*	F: 5′-TGC TGC TAC TCA TTC ACT GGC-3′
	R: 5′-CCT TAT TGG GGT CAG CAC AG-3′
*Kir6.1*	F: 5′-AAA GGC ATC ACG GAG AAG AGT-3′
	R: 5′-TGG AGA AGA GAA ACG CAG AAG-3′
*Kir6.2*	F: 5′-AGC ATC CAC TCC TTT TCG TCT-3′
	R: 5′-GCT TGC TGA AGA TGA GGG TTT-3′
*SUR1*	F: 5′-TTT TGG ATG ACC CTT TCT CG-3′
	R: 5′-AGT GTC CCC TCC CTC TGA AT-3′
*SUR2*	F: 5′-TTT GCC TCT CTG TCT CTC TTC C-3′
	R: 5′-CTG CTT CCT GTT TAT CGG TTT T-3′

F, forward; R, reverse.
